# Using illusions to understand hallucinations: differences in perceptual performances on illusory figures may underscore specific visuoperceptual impairments in Parkinson’s disease

**DOI:** 10.3389/fnins.2023.1256224

**Published:** 2023-12-06

**Authors:** Alberto Cucca, Claudia Virginia Manara, Mauro Catalan, Marco Liccari, Lucia Antonutti, Tiziana Maria Isabella Lombardo, Valentina Cenacchi, Sophie Rangan, Serena Mingolo, Carmelo Crisafulli, Franca Dore, Mauro Murgia, Tiziano Agostini, Paolo Manganotti

**Affiliations:** ^1^Department of Life Sciences, University of Trieste, Trieste, Italy; ^2^Department of Neurology, New York University Grossman School of Medicine, New York, NY, United States; ^3^Neurology Clinic, Department of Medicine, Surgery and Health Sciences, University of Trieste, Trieste, Italy; ^4^Nuclear Medicine, Imaging Diagnostic Department University Hospital and Health Services of Trieste, Trieste, Italy

**Keywords:** hallucinations, illusions, perception, Parkinson’s disease, visual cognition

## Abstract

Visual hallucinations are prevalent, potentially disabling symptoms of Parkinson’s Disease. Multiple impairments in bottom-up sensory processing and top-down perceptual modulation are implicated in the pathophysiology of these phenomena. In healthy individuals, visual illusions are elicited by illusory figures through parametric manipulations of geometrical configurations, contrast, color, or spatial relationships between stimuli. These illusory percepts provide insight on the physiologic processes subserving conscious and unconscious perception. In this exploratory, cross-sectional, controlled study, perceptual performance on illusory figures was assessed on 11 PD patients with hallucinations, 10 non-hallucinating PD patients, and 10 age-matched healthy individuals. In order to characterize potential neural substrates of perceptual performances, patients’ brain metabolic patterns on FDG PET were also analyzed. Illusions relying on attentional modulation and global perception were attenuated in PD patients without hallucinations. This pattern was no longer recognizable in hallucinating patients. Conversely, illusory effects normally counteracted by figure to background segregation and overlapping figures recognition were enhanced in PD patients with hallucinations. FDG PET findings further suggest that perceptual differences between PD patients might be linked to abnormal top-down perceptual modulation.

## 1 Introduction

Recurrent illusions and visual hallucinations are prevalent and poorly understood non-motor symptoms of Parkinson’s disease (PD) (Diederich et al., 2009). Hallucinations are sensory perceptions occurring without external stimulation of the relevant sensory organ. This definition allows to differentiate them from illusions, in which an external stimulus is perceived, but misinterpreted. Approximately 40% of patients with PD report visual hallucinations during the course of the disease (Fénelon et al., 2000). The risk for hallucinations increases with the disease progression, with a point prevalence reaching 74% of patients over a 20 years long follow-up (Lenka et al., 2019). Common illusions experienced by PD patients include feelings of motion (kinetopsia), abnormal perceptions of distance (teleo/pelopsia), abnormal perceptions of size (macro/micropsia), and object misidentifications (Nishio et al., 2018). Pareidolias are also frequently experienced by patients with PD, and they can be defined as illusory percepts due to an erroneous attribution of meaningful content to random or ambiguous visual patterns (Kurumada et al., 2021). Visual hallucinations are, in turn, categorized into “simple” versus “complex” hallucinations. Simple hallucinations are characterized by the absence of recognizable shape. Complex hallucinations usually involve animate characters engaged in scenes of routine life, like pets, children playing in the background, or family members visiting the patient (Barnes and David, 2001). Growing evidence suggests that in PD, complex hallucinations are driven by an impaired bottom-up processing of sensory information, in combination with abnormal top-down perceptual modulation (Lenka et al., 2015; Lefebvre et al., 2016; Marques et al., 2022). Patients with PD may exhibit impairments on various visuospatial functions, including dynamic shape perception, orientation judgment, stereopsis, and motion perception (Koerts et al., 2010; Weil et al., 2016; Montagnese et al., 2022). Figure to background segregation and perceptual judgement of overlapping figures may also be affected (Mosimann et al., 2004). Furthermore, in these patients, visual recognition strategies may involve an overreliance on local features analysis, resulting in poorer performances in matching judgments on various shape recognition tasks (Kerai et al., 2012). When the quality and reliability of afferent visual information is affected, the compensatory recruitment of higher processing areas boosting selective attention, suppressing distraction, and retrieving relevant priors stored in visual memory is likely to occur (Kveraga et al., 2007). However, top-down perceptual functions are not optimized to systematically fill the gap of perceptual ambiguities resulting from poor quality sensory information. As a result, erroneous percepts with growing layers of complexity may occur (Thomas et al., 2022).

Different from pathological illusions and hallucinations in PD, in experimental psychology perceptual illusions are phenomena in which healthy individuals consistently misperceive stimuli (Gregory, 1968; Coren et al., 1976). These illusions are inherent to the physiological properties of the human visual system and, as such, they do not hold pathological relevance *per se*. Rather, these misinterpretations highlight discrepancies between the physical properties of external information and its final percept, thus providing valuable information on the physiologic processes subserving perception (Coren and Girgus, 1978). To avoid ambiguities, in this article, the term “illusory figures” will be used to refer to configurations of visual stimuli purposefully eliciting illusions in healthy individuals, whereas the term “illusions” will be used to denote the perceptual experience of observers exposed to such figures. In the present study, we aimed to investigate the perceptual performance of non-demented PD patients on illusory figures, with and without a history of hallucinations, and to compare it with that of age-matched healthy individuals. To this end, two illusory figures were selected: the Delboeuf illusion and the size shrinkage due to amodal completion. The Delboeuf illusion was chosen to explore potential differences in perceptual performance related to spatial scaling (size contrast), perceptual grouping (assimilation), and attentional modulation (judgement order effect). The illusion of size shrinkage was selected to explore potential differences in perceptual performances related to amodal completion, perceptual filling and overlapping figures judgement. Potential neural substrates of perceptual performances in both hallucinating and non-hallucinating PD patients were further characterized by means of F-18 fluoro-deoxy-glucose positron emission tomography [(Gregory, 1968) FDG PET]. Final perception results from the dynamic interplay between mechanisms preventing the misinterpretation of sensory information (e.g., perceptual constancies) and mechanisms generating illusory biases. In this setting, different profiles of vulnerability to illusions can be hypothesized in PD, depending on patients’ visuoperceptual impairment. An increased vulnerability to illusions should be observed when relevant protective mechanisms are targeted by the disease. Conversely, a paradoxical profile of decreased vulnerability should be expected when mechanisms normally driving the occurrence of illusions are affected by the underlying neurodegenerative process.

## 2 Materials and methods

### 2.1 Study design

Observational, cross-sectional, controlled, exploratory study conducted on three groups of age-matched individuals: PD patients with history of complex hallucinations (PD_Hal), PD patients with no history of hallucinations (PD_NonHal), and healthy controls (HC). Main inclusion criteria were a Montreal Cognitive Assessment (MoCA) corrected score ≥ 24 and, for PD patients, a clinically established diagnosis of PD according to the United Kingdom Parkinson’s Disease Society (UKPDS) Brain Bank criteria (Calne et al., 1992). Main exclusion criteria were a history of clinically significant ocular pathology or ophthalmic disease, and impaired visual acuity as indicated by a Snellen chart acuity test <20/20, despite potential correction. Specific exclusion criteria for PD patients included recent changes in dopaminergic medications, unpredictable motor fluctuations, psychosis, delirium, or any contraindication to undergo (Gregory, 1968) FDG PET study. Participants were prescreened telephonically to determine potential eligibility. On Visit 1, eligibility criteria were reviewed, and PD patients were categorized as PD_Hal and PD_NonHal based on the score of their University of Miami Parkinson’s disease Hallucinations Questionnaire (UM-PDHQ). The UM-PDHQ is 20-item clinician-administered questionnaire consisting of two groups of questions: a quantitative group of 6 questions assessing modality, frequency, duration, insight and emotional burden, and a qualitative group consisting of 14 questions assessing clinical phenomenology as well as the potential association with dopaminergic medications and concomitant ocular abnormalities (Papapetropoulos et al., 2008). For the present study, an Italian translation of the UMPDHQ was developed by the corresponding author (see in Supplementary material). Patients scoring ≥1 were categorized as PD_Hal, whereas patients scoring 0 were categorized as PD_NonHal. UM-PDHQ was chosen to determine patients’ hallucinatory status in light of its relatively higher sensitivity, accuracy in describing disease-specific hallucinatory percepts, and nominal time requirements for its administration. Upon successful verification of eligibility, participants were scheduled with Visit 2, when the remaining experimental procedures took place. In PD patients experiencing motor fluctuations, all assessments on Visit 2 were conducted in the ON therapeutic state in order to minimize fatigue and potential discomfort. Study personnel involved in the assessments of Visit 2 was kept blind to the specific PD group allocation.

### 2.2 Study setting and participants

The study was carried out between February and September 2022 at the Neurology Clinic of Cattinara Teaching Hospital in Trieste, in collaboration with the Department of Life Sciences of the University of Trieste, Italy. Thirty-three subjects were consecutively screened, and 31 of them were deemed eligible to participate in the study. Informed consent for data collection was undersigned by all participants, and the study was approved by the local institution review board (Comitato Etico Unico Regionale FVG, CEUR). All experimental activities were performed in accordance with relevant regulations and in compliance with the Declaration of Helsinki.

#### 2.2.1 Illusory figures: apparatus

Two sets of illusory figures were used in the present experiments: the Delboeuf illusion, and the size shrinkage of amodal completion. For each illusion, a set of stimuli was created by keeping constant one part of the configuration while systematically manipulating one variable of the second part of the configuration. For the Delboeuf illusion, the diameter of the target enclosed by the larger inducer (on the right side) was manipulated ranging from 0.95° to 2.29° of visual angle (with a variation of 0.09° for each figure); the size of the inner disk on the left was constant (1.09°). For the size shrinkage of amodal completion, the width of the non-occluded square (on the right side) was manipulated ranging from 4.39° to 7.81° of visual angle (with a variation of 0.19° for each figure); the size of the occluded square on the left was constant (5.72°). Stimuli were generated using a vector graphics editor (Inkscape). The experiment was programmed through an open source software package written in Python (PsychoPy). Illusory figures were administered with participants sitting in front of a computer screen of 31.5 × 54 cm placed in front of them on a distance of approximately 50 cm. A five-buttons response box was used to collect responses, using the extreme left and right keys for responses.

#### 2.2.2 Illusory figures: procedures

**Figure 1:**
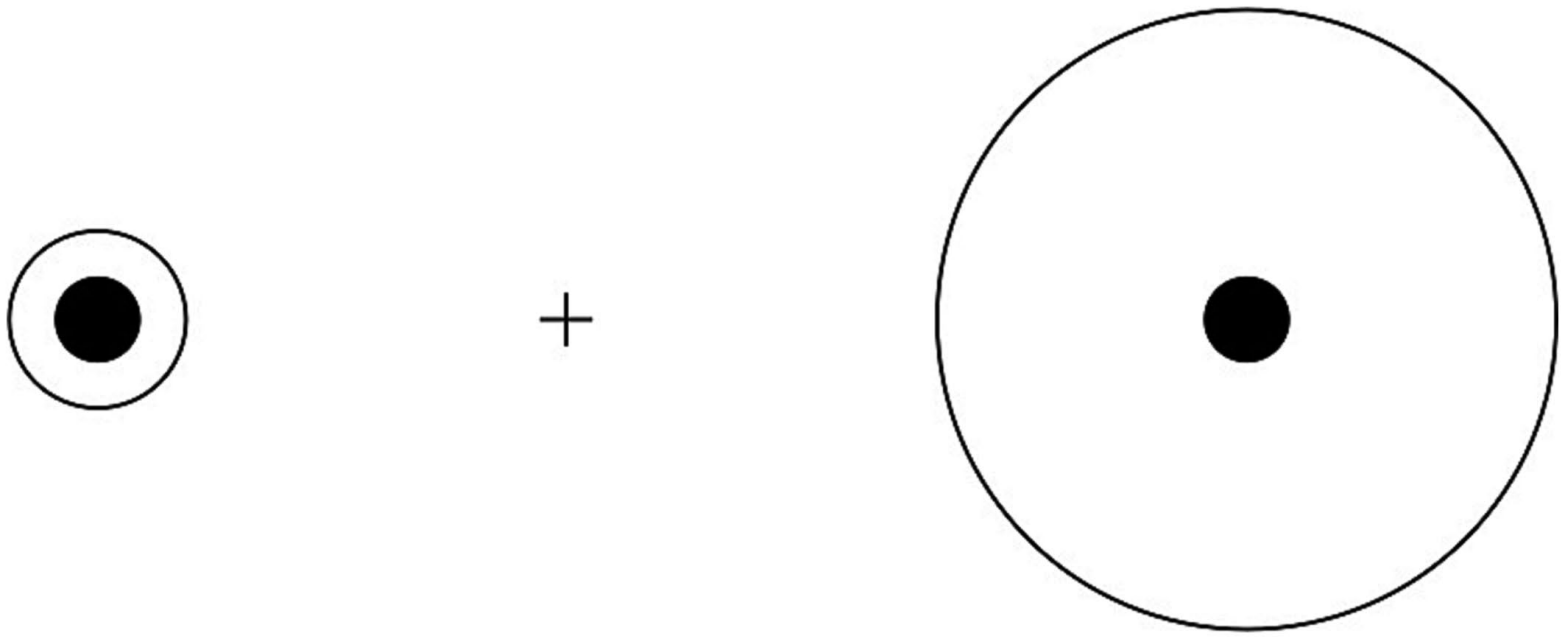
Size distortions in Delboeuf illusion: when the outer circle is slightly larger than the inner circle (left), the latter is overestimated due to illusory assimilation; when the outer circle is considerably larger than the inner circle (right), the latter is underestimated due to size contrast.

**Figure 2:**
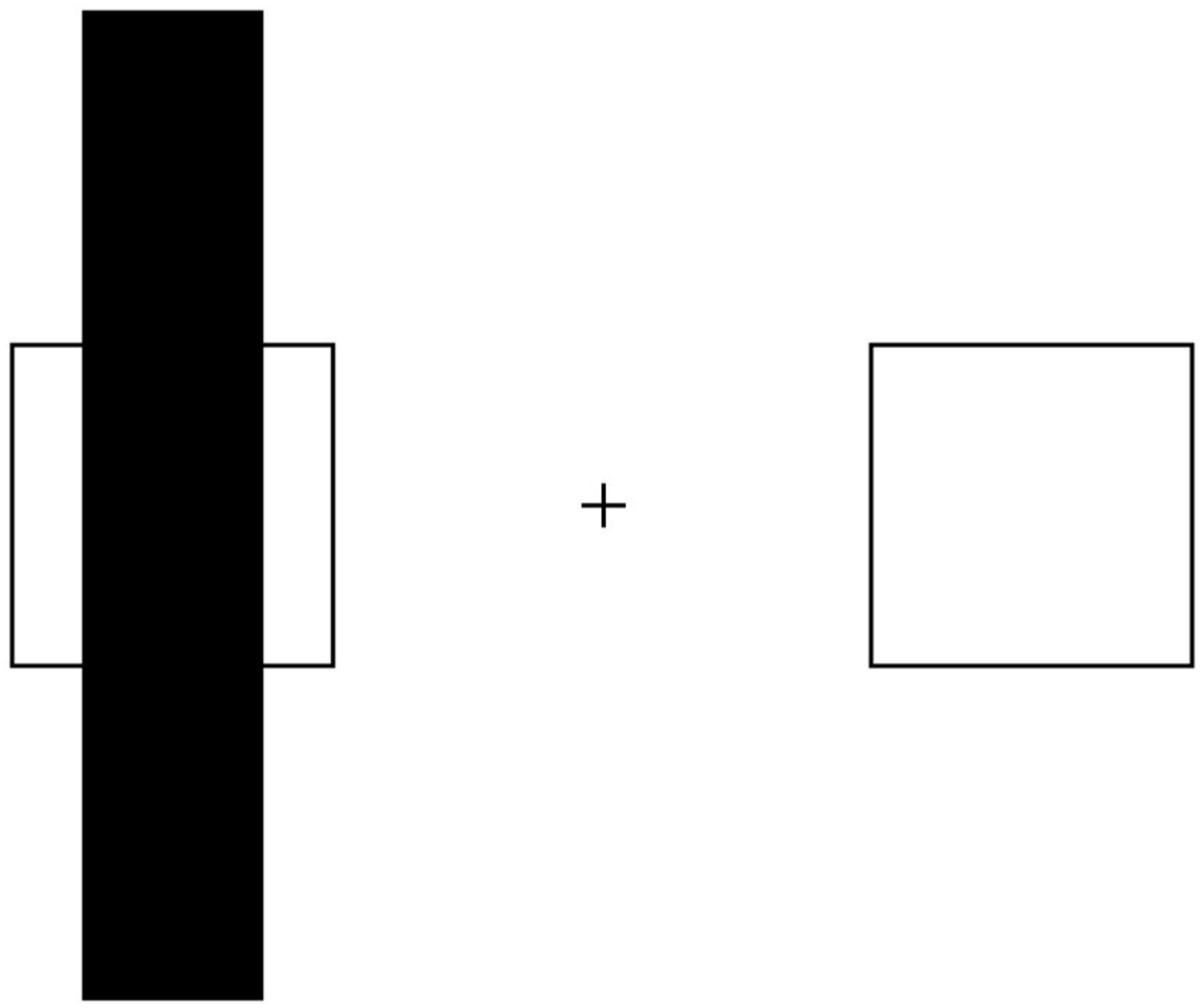
Illusory shrinkage of amodally completed objects: the occluded square can be erroneously perceived as smaller than its non-occluded copy.

Perceptual performance on illusory figures was assessed in PD patients without hallucinations, hallucinating PD patients, and healthy controls. Participants were exposed to a set of illusory figures (one by one) and were asked to provide their answer by pressing the corresponding key on the response box in front of them, using the left hand for pressing the left key and right hand for the right key. When performing the task related to the Delboeuf illusion, participants were asked to answer the following question: “*can you tell me which one of the two inner circles is larger?*” (Figure 1). Similarly, when performing the task relevant to the amodal completion illusion, participants were asked to answer the following question: “*can you tell me which one of the two squares is larger?*” (Figure 2). In both tasks, participants were instructed to press the left key when the left target was perceived larger than the right one, and the right key when the right target was perceived larger. A familiarization session was run before starting the experiment for each perceptual task. No specific time constraints were given, but participants were prompted to undergo the tasks in a timely fashion and at best of their capability. A staircase method was employed, with the initial stimulus always eliciting a perceptually obvious judgement, either a maximally amplified illusion or its opposite perceptual effect. The perceptual variable relevant to each illusion was then systematically manipulated by showing figures that progressively reduced the effect until the participant’s initial perceptual judgement was reversed and the reversal was confirmed in a subsequent trial. At this point, a new set of stimuli was administered, this time starting from the opposite end of the range of the stimuli. The same procedure was repeated four times for each illusion, with the starting condition being randomized across trials. For each set of stimuli, the average value of the two figures before and after the reversal was calculated. This value represents the subject’s point of subjective equality (PSE), i.e., the value of comparison stimulus equally likely to be judged higher or lower than that of the standard stimulus. An average PSE close to the point of physical equality was taken as evidence for an accurate perceptual performance. Conversely, the greater the difference between PSE and point of physical equality, the more vulnerable participants were deemed towards the relevant illusory effect. The overall time for the administration of both illusory figures was approximately 10 min.

### 2.3 Clinical assessments

Clinical assessments were performed exclusively on PD patients by neurologists with expertise on Movement Disorders (AC, ML, and TL). Disease burden and clinical severity were assessed by means of MDS-UPDRS, in its four sections exploring mentation, behavior and mood, activities of daily living (ADL), motor severity, and complications of therapy, respectively (Goetz et al., 2008). A specific sub-score was further derived for each patient by pooling together scores on item 1 and 9–13 of MDS-UPDRS Part III in order to explore potential differences in terms of axial impairment between PD_NonHal and PD_Hal. The 39 item PD questionnaire (PDQ-39) was administered to assess patient’s perceived quality of life and functional independence (Peto et al., 1995). Patient’s pharmacological profile was characterized in terms of levodopa equivalent dose (LEDD) and dopamine-agonist equivalent dose (DA-LED) as in Tomlinson et al. (2010).

### 2.4 Study of brain metabolism with F-18 fluoro-deoxy-glucose positron emission tomography

Regional cerebral metabolic rate of glucose utilization was measured using FDG PET in PD patients exclusively (Gregory, 1968). All patients fasted for more than 6 h before the scan and were injected with a mean FDG dose of 200 MBq intravenously 40 min prior to scanning. To minimize the effects of external stimuli during the 40 min FDG-uptake period, subjects were confined in a quiet room. Scans were obtained with patients under resting conditions, wearing eye masks. PET images were obtained using a PET/TC Discovery MI DR scanner (G.E. Healthcare) for 8 min. Image reconstruction was performed using an ordered subset expectation maximization and 32 subsets and a 5-iteration reconstruction algorithm and displayed in a 128 × 128 matrix (pixel size 2.35 mm). Multiple automated approaches for alignment to the database were employed, including linear affine registration to account for global position and scaling differences as well as a deformable registration algorithm to allow for localized adjustments. On each of the spatially and globally normalized images of patients, *Z*-scores were obtained through a dedicated post-processing software (Cortex Suite, G.E.) for each of the following region of interest (RoI), bilaterally: lateral prefrontal cortex (LPFC), medial prefrontal cortex (MPFC), superior parietal cortex (SPC), inferior parietal cortex (IPC), lateral temporal cortex (LTC), mesial temporal cortex (MTC), lateral occipital cortex (LOC), precuneus, and primary visual cortex (PVC) as in Tzourio-Mazoyer et al. (2002). Positive *Z* scores indicated relative hypermetabolism and negative *Z* scores indicated relative hypometabolism. Semiquantitative assessments were obtained through and reviewed by expert nuclear medicine physicians (Dinoto et al., 2021).

## 3 Statistics

Participants’ PSE was calculated from each sequence of illusory figures by averaging the value of the stimulus before and after the inversion, considering the dimension being manipulated (i.e., the degree of visual angle). A set of one-way ANOVAs were conducted for the individual sets of each illusory figure; the dependent variable was the PSE of each individual series. Tuckey’s correction (Honestly Significant Difference) was applied in post-hoc tests. The threshold value for significance was set at *p* < 0.05. Clinical variables across the two PD groups were compared with two-tailed *t*-test for unequal variance. Statistical significance threshold was set at *α* = 0.05. For comparisons that did not meet the criteria for using parametric tests, the corresponding non-parametric tests were utilized. Between group differences in brain metabolic patterns on FDG PET were analyzed by calculating the values of mean regional abnormalities in relative regional cerebral glucose metabolic rate for each RoI. Statistical analyses were performed by means of *t* testing for the two PD groups across each region, with significance level set at *p* < 0.05.

## 4 Results

### 4.1 Demographic and general characteristics

Ten healthy subjects and 21 PD patients were enrolled in the study. On Visit 1, 10 PD patients with a UMPDHQ score of 0, and 11 PD patients with a UMPDHQ score ≥1 were assigned to the PD_NonHal and the PD_Hal group, respectively. Demographic characteristics and clinical features of study population are summarized in Table 1. Subjects in the three groups were comparable in terms of age, gender distribution, and general cognition as assessed by MoCA. Comparatively longer education was observed in HC and PD_Hal as compared to PD_NonHal.

**Table 1:** Demographics and general clinical features.

	PD_Hal	PD_NonHal	HC	*p* value^a^	*p* value^b^	*p* value^c^
Age (yrs); mean (±SD)	65.8 (±7.68)	69.3 (±6.22)	69.2 (±5.16)	0.27	0.255	0.969
Gender (F:M)	3:8	2:8	3:7	–	–	–
Education (yrs); mean (±SD)	14.63 (±3.41)	11.1 (±3.35)	15.1 (±3.07)	0.027*	0.748	0.012*
MoCA_raw; mean (±SD)	26.68 (±2.51)	27 (±1.94)	26.9 (±2.51)	0.36	0.5	0.51
MoCA_ corrected; mean (±SD)	26.72 (±2.53)	28.3 (±1.34)	27.2 (±2.15)	0.127	0.651	0.131
Disease Duration (yrs); mean (±SD)	7.64 (±5.02)	5.4 (±2.67)	N/A	0.387	–	–
Hoehn and Yahr	3 HY1, 8 HY2	3 HY1, 5 HY2, 2 HY3	N/A	–	–	–
UMPDHQ, questions 1–6 mean subscore (±SD) (min. 0 max. 14)	6.72 (±3.10)	0	–	–	–	–
LEDD; mean (±SD)	683 (±268)	633 (±296)	N/A	0.973	–	–
DA-LEDD; mean (±SD)	218.18 (±76)	127.5 (±141.64)	N/A	0.152	–	–
MDS-UPDRS I; mean (±SD)	12.27 (±5.53)	6.88 (±3.1)	N/A	0.024^≠^	–	–
MDS-UPDRS-II; mean (±SD)	9.63 (±3.41)	4 (±3.35)	N/A	0.001*	–	–
MDS-UPDRS III; mean (±SD)	34.72 (±11.67)	27 (±11.66)	N/A	0.14	–	–
UPDRS III_axial; mean (±SD)	5.63 (±2.37)	3.08 (±2.09)	N/A	0.08^≠^	–	–
MDS-UPDRS IV; mean (±SD)	2.18 (±2.27)	3.01 (±1.88)	N/A	0.80	–	–
MDS-UPDRS Total; mean (±SD)	58.81 (±15.36)	39 (±17.95)	N/A	0.01*	–	–
PDQ39_total; mean (±SD)	36.72 (±14.17)	19.77 (±8.49)	N/A	0.020*	–	–
PDQ39_mobility; mean (±SD)	7.72 (±4.94)	3 (±3.04)	N/A	0.028^≠^	–	–
PDQ39_ADL; mean (±SD)	6.18 (±3.57)	2.7 (±2.04)	N/A	0.025*	–	–
PDQ39_Stigma; mean (±SD)	2.63 (±2.24)	1.66 (±1.65)	N/A	0.296	–	–
PDQ39_Cognition; mean (±SD)	6.18 (±3.37)	3.22 (±1.71)	N/A	0.028*	–	–

Between group comparison **(a)**, PD_Hal vs. PD_NonHal; **(b)**, PD_Hal vs. HC; **(c)**, PD_NonHal vs. HC; *p* < 0.05 (*) by 2-tailed independent *t*-test; (^≠^) by Mann–Whitney/Wilcoxon test. MoCA, Montreal Cognitive Assessment; UMPDHQ, The University of Miami Parkinson’s Disease Hallucinations Questionnaire; LEDD, Levodopa Equivalent Dose; DA-LEDD, dopamine agonist adjusted equivalent dose; MDS-UPDRS, Movement Disorders Society United Parkinson’s Disease Rating Scale; PDQ39, Parkinson’s Disease Questionnaire 39; N/A, non-applicable.

### 4.2 Perceptual performances on illusory figures

**Figure 3:**
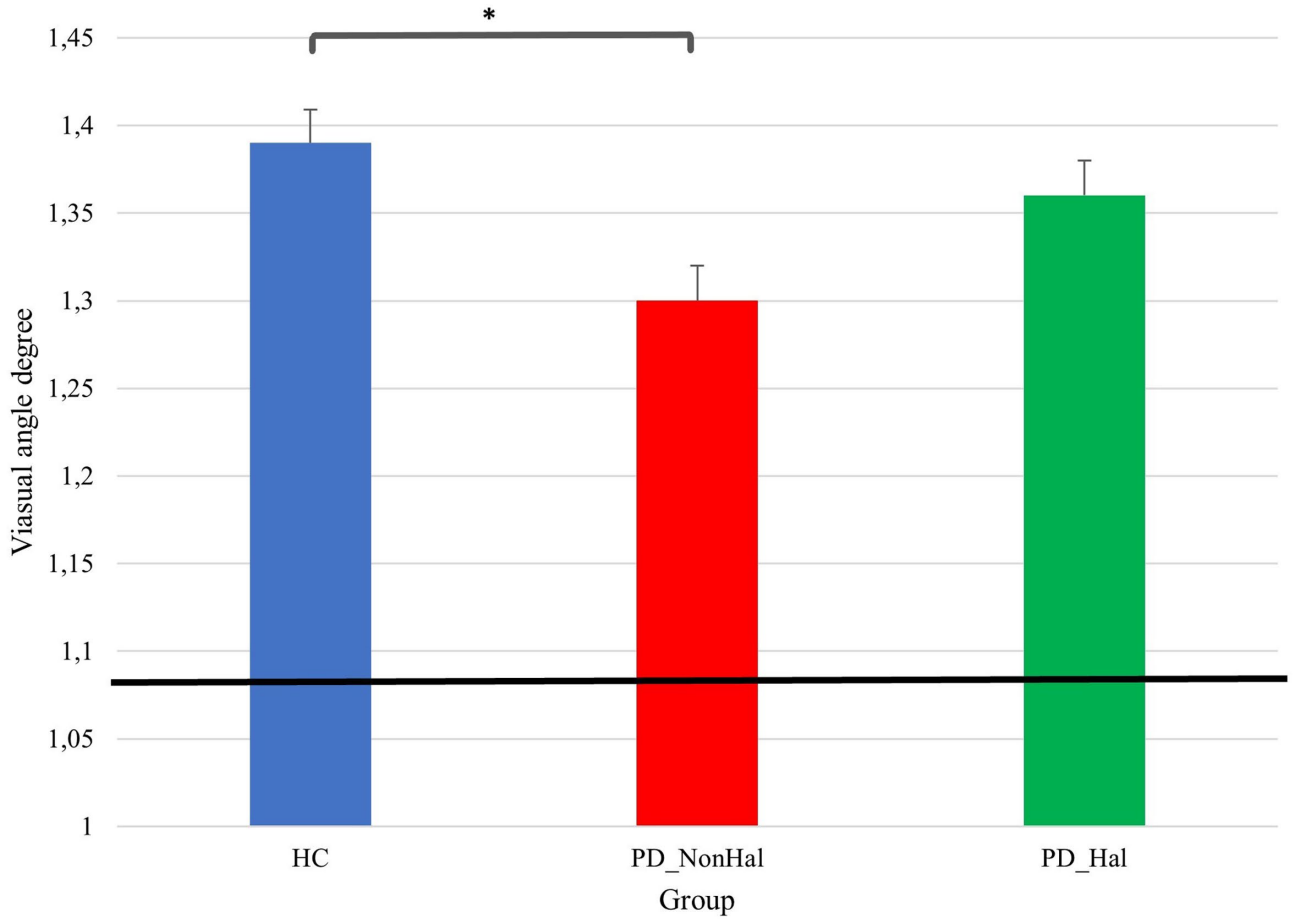
Perceptual performance on size estimation task: mean PSE (vertical bars) vs point of physical equality (horizontal black bar) across the three groups. In this illusion, the lower the visual angle degree, the better the performance, as this is closer to the point of physical equality, *p < 0.05 by one-way ANOVA.

**Figure 4:**
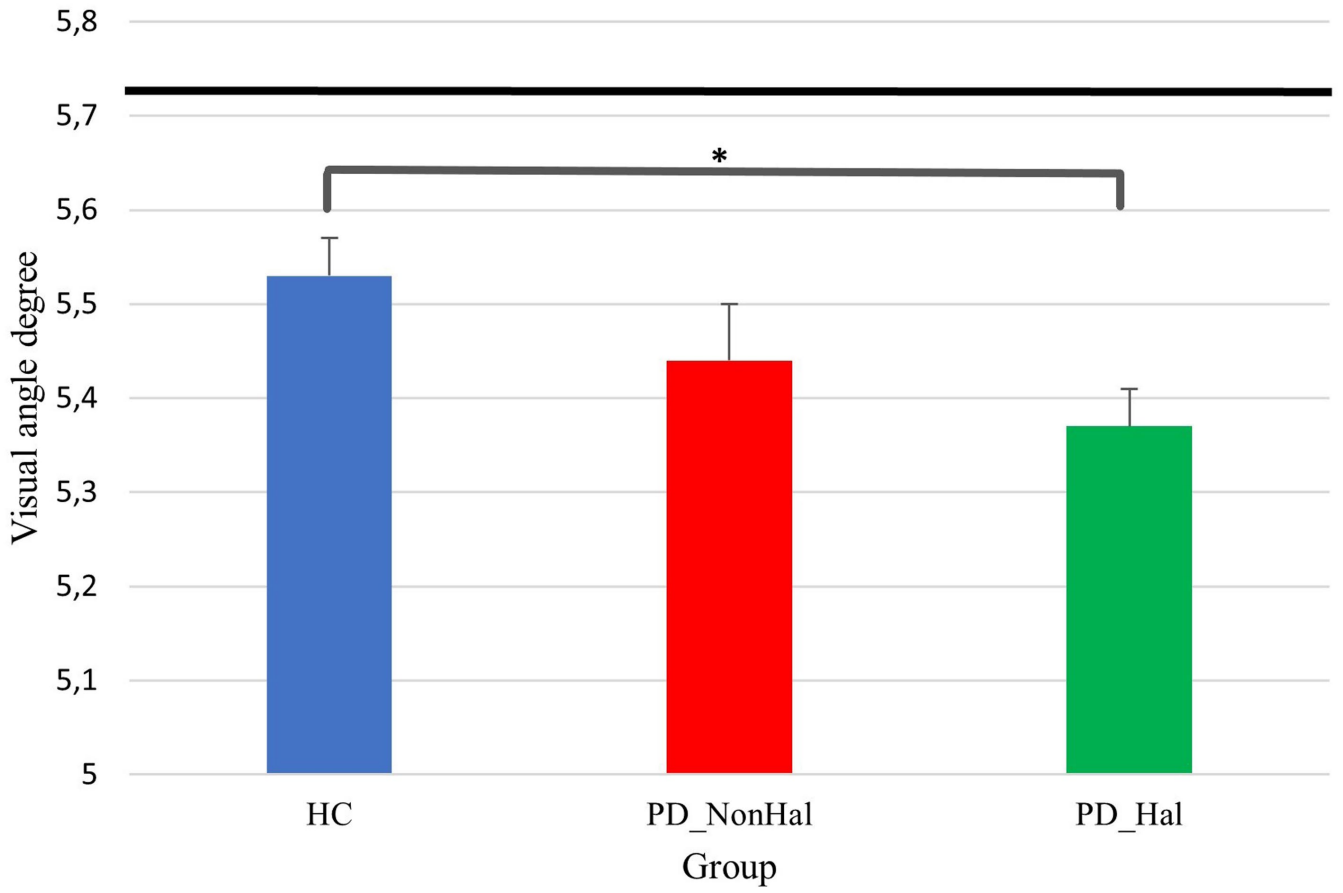
Perceptual performance on amodal completion: mean PSE (vertical bars) Vs point of physical equality (horizontal black bar) across the three groups. In this illusion, the higher the visual angle degree, the better the performance, as this is closer to the point of physical equality. *p < 0.05 by one-way ANOVA.

Differences in perceptual performances on Delboeuf illusion and amodal completion illusions between PD_NonHal, PD_Hal and HC are highlighted on Figures 3 and 4, respectively. On the size estimation task of the Delboeuf illusion, PD patients without hallucinations performed significantly better than healthy controls (physical equality: 1.09°; mean PSE in PD_NonHal 1.30°; mean PSE in HC: 1.39°; *p* < 0.05). Non-hallucinating patients also performed better than hallucinators, though not significantly so (mean PSE in PD_Hal 1.36°). Performance of hallucinating patients was substantially similar to healthy controls.

Perceptual performance on the amodal completion illusion was significantly worse in hallucinating PD patients as compared to healthy controls (physical equality: 5.72°; mean PSE in PD_Hal: 5.36°; mean PSE in HC: 5.53°; *p* < 0.05). Hallucinators also performed poorly when compared to non-hallucinating PD patients, though not significantly so (mean PSE in PD_NonHal: 5.44°).

### 4.3 Clinical features

General Motor impairment as assessed by MDS-UPDRS part III was comparable between PD patients with and without hallucinations, but a trend towards a worse axial involvement was found in PD_Hal as compared to PD_NonHal (mean UPDRS Part III axial subscore: 5.63 ± 2.37 vs 3.08 ± 2.09; *p* = 0.08). Patients in the PD_Hal group showed significantly worse quality of life and perceived functionality in various ADL compared to PD_NonHal, as indicated by scores in PDQ-39 (total score, as well as mobility, ADL and cognition subscores), MDS-UPDRS part I, and MDS-UPDRS part II. No significant differences in LEDD and DA-LEDD emerged between the two PD groups.

All hallucinating patients reported full insight on their hallucinations. In the majority of hallucinating patients (7/11, i.e., 64% of the sample), the frequency of hallucinations was lower than once a week. Pure visual hallucinations were reported by 45% of hallucinating patients (5/11), while in the remaining cases hallucinations occurred in multiple sensory modalities involving visual, acoustic, tactile, and olphactory percepts. Fully formed visual hallucinations were reported by 82% of PD_Hal (9/11). In these cases, the content of hallucinations always involved animated perceptions, either anthropomorphic or zoomorphic (e.g., relatives, significant others, pets, or wild animals).

### 4.4 Brain metabolic patterns

Mean *Z*-scores for each RoI are summarized in Table 2. Both groups showed similar hypometabolic patterns involving the following regions: SPC, LPC, LTC, MTC, LOC and PVC, bilaterally. Different patterns between the two groups emerged at the level of left MPFC. Here, hallucinating PD patients showed a relative hypermetabolism compared to a mild hypometabolism of non-hallucinating patients, as indicated by higher *Z* scores in the relevant RoI (0.478 ± 1.821 vs −1.602 ± 1.909; *p* = 0.033 by independent *t*-test).

**Table 2:** Mean *Z* scores obtained for each designated RoI in PD patients without (PD_NonHal) and with history of visual hallucinations (PD_Hal).

RoI	PD_NonHal mean *Z*-score (±SD)	PD_Hal mean *Z*-score (±SD)	*p* value^a^
LPFC_R	−1.032 (±0.823)	−0.42 (±1.672)	0.245
LPFC_L	−1.48 (±1,296)	−1.222 (±1.055)	0.65
MPFC_R	−1.18 (±1.476)	0.2 (±1.782)	0.106
MPFC_L	−1.602 (±1.903)	0.478 (±1.821)	0.033*
SPC_R	−3.707 (±1.938)	−2.461 (±1.711)	0.171
SPC_L	−2.845 (±1.752)	−2.499 (±1.658)	0.677
IPC_R	−3.254 (±2.326)	−3.509 (±2.212)	0.636
IPC_L	−3.955 (±1.878)	−3.705 (±2.182)	0.701
LTC_R	−1.855 (±0.574)	−1.758 (±1.267)	0.851
MTC_L	−2.08 (±1.358)	−1.944 (±1.647)	0.858
TMC_R	0.984 (±1.251)	1.609 (±1.814)	0.438
TMC_L	1.231 (±1.592)	1.804 (±1.830)	0.506
LOC_R	−3.995 (±1.878)	−4.068 (±2.66)	0.95
LOC_L	−3.738 (±0.988)	−4.263 (±2.88)	0.651
Precuneus_R	4.155 (±1.955)	−2.832 (±2.256)	0.221
Precuneus_L	−3.254 (±2.326)	−0.884 (±2.635)	0.069
PVC_R	−1.83 (±1.031)	−2.321 (±1–101)	0.358
PVC_L	−1.765 (±1.045)	−2.53 (±1.053)	0.151

(a): between-group comparison by 2-tailed independent *t*-test; (*): *p* < 0.05. LPFC, lateral prefrontal cortex; MPFC, medial prefrontal cortex; SPC, superior parietal cortex; IPC, inferior parietal cortex; LTC, lateral temporal cortex; MTC, mesial temporal cortex; LOC, lateral occipital cortex; PVC, primary visual cortex; R, right-side; L, left-side.

## 5 Discussion

In the past years, growing efforts were dedicated to characterize impairments in visuoperceptual functions potentially linked to the onset of visual hallucinations in PD. Recently, patients with PD and hallucinations were reported to make a significantly higher number of pareidolic errors as compared to non-hallucinating patients in a dedicated 20-items neuropsychological task, suggesting that impaired visuospatial abilities may indeed play a central role in the pathophysiology of hallucinatory percepts (Turner and Rodriguez-Porcel, 2023). The most striking finding of this study was that visuospatial deficits in PD appear to differentially affect systematic perceptual biases driving illusions, resulting in variable performances on computer-generated illusory figures. The Delboeuf illusion is a visual phenomenon first described by the Belgian philosopher Franz Joseph Delboeuf occurring when two circles (test figures) of equal radius are presented next to each other and surrounded by concentric circles (inducers) of different radii. In this illusion, the size of the test figure is overestimated or underestimated depending on the size of its inducer (Parrish et al., 2015). If the inducer is only slightly larger than the target, the latter will be assimilated and hereby perceived bigger than its real size, a phenomenon known as “assimilation illusion” (McCarthy et al., 2013). According to our results, PD patients without hallucinations exhibit a more accurate perceptual performance than healthy controls in the Delboeuf size estimation task, as indicated by a significantly smaller gap between their mean PSA and point of physical equality. We interpreted this paradoxical resistance to the assimilation illusion as a result of differences in the effect of judgement order. This is a well-known phenomenon whereby the magnitude of the overestimation distortion is reduced when the target is attended before the inducer due to the persistence of spatial scaling from the prioritized stimulus (Kawahara et al., 2007). Notably, when performing our size estimation task, subjects were asked to attend the inner circles prior to the inducers. In this setting, a further attentional effort is required to integrate the new spatial scale of the inducer, thus reducing the strength of the illusion. PD patients are known to exhibit a pattern of impaired visual attention characterized by overly rigid selective attention and poor set shifting (Gauntlett-Gilbert et al., 1999; Botha and Carr, 2012; Fallon et al., 2016). Furthermore, in these patients visual exploration strategies heavily rely on local rather than global visual exploration (Matsumoto et al., 2013). An impaired attentional modulation with a compressed global representation of illusory figures might have resulted in an attenuation of the assimilation distortion. Interestingly, this “protective effect” was no longer recognizable in hallucinating patients, thus suggesting a potential disruptive effect of hallucinations on this pattern.

A different perceptual profile emerged on the amodal completion illusion. Here, a greater vulnerability towards the illusory shrinkage effect was found in hallucinating PD patients, as indicated by a significantly greater gap between mean PSE and point of physical equality compared to healthy controls. Although perceptual differences between PD_Hal and PD_NonHal did not meet statistical significance, a general pattern of increased vulnerability clearly emerged across the three groups whereby perceptual performance on this illusory figure was poorer in non-hallucinating PD patients compared to controls, and it further deteriorated in PD hallucinators. The illusory shrinking is a well-known perceptual distortion arising when two modally visible elements of an occluded object are perceived as a unitary object. In a classic example of this distortion, a square lying behind an occluding rectangle is perceived as smaller than its non-occluded copy. Different hypotheses have been formulated to explain the shrinkage illusion. Kanizsa famously noted that the perceived extension of surfaces depends only partially on their actual geometric extension, as the size representation of objects is influenced by the low intensity and homogeneity of the stimulation of the occluded surface (Kanizsa, 1975). In this setting, observers disregard the perceptual evidence supporting the existence of two same-sized squares by erroneously perceiving the amodally completed copy as shrunk (Gerbino, 2020). The ability to perform figure to background segregation is key to accurate perceptual decisions regarding overlapping figures, as this function enables observers to attend each element of the visual scene separately, hence reaching accurate perceptual decisions (Shoji et al., 2014). Interestingly, PD patients were previously found to perform poorly on modified versions of Poppelreuter-Ghent’s overlapping figure tests, where the disentanglement of overlapping objects requires both intact figure to background segregation and the ability to explore each figure across various spatial configurations (Ishioka et al., 2011). Furthermore, a specific impairment in the ability of PD patients to isolate discrete visual features when embedded into complex sensory patterns was recently found in PD patients (Cucca et al., 2021). We hypothesized that the increased vulnerability to the shrinkage illusion of amodal completion in PD patients might be related to the pathological involvement of cortical areas involved in perceptual grouping, overlapping figures judgement, and figure to background segregation. According to computational models and recent functional connectivity studies, key components of these processes such as iso-feature detection and iso-feature suppression rely on feedback loops projecting from higher cortical areas within the frontal and dorsolateral prefrontal cortices towards the primary visual cortex (Murray et al., 2004). The pathological involvement of these high order perceptual areas in hallucinating patients might explain their greater vulnerability towards this particular illusory bias. Indeed, a key role played by abnormalities affecting top-down attentional modulation in hallucinating PD patients was further suggested by our FDG PET findings. Here, a pattern of relative hypermetabolism in prefrontal cortical regions concerned with attentional modulation of upcoming sensory information was observed, a finding in agreement with previous literature reporting compensatory activation of anterior cortical networks, usually in the setting of hypometabolism affecting posterior areas such as the parietal and occipital cortices (Nagano-Saito et al., 2004; Uchiyama et al., 2015; Tan et al., 2023).

From a clinical viewpoint, PD patients with and without visual hallucinations were comparable in terms of disease severity, disease duration, general cognition, and overall motor impairment. These findings strongly support the primary perceptual nature of the observed differences in performance on illusory figures. Furthermore, the inclusion of PD patients without evidence of cognitive impairment or history of psychosis allowed for an accurate characterization of their perceptual functions while minimizing potential confounders due to comorbid cognitive or psychiatric factors. When compared to non-hallucinators, hallucinating PD patients exhibited a trend towards a worse axial involvement. This finding might have also contributed to worse performances in their activities of daily living. Indeed, PD patients with a history of visual hallucinations perceived a greater functional impairment in multiple domains of daily living as compared to PD patients without hallucinations, despite a comparable overall severity of the underlying disease. These findings support the potentially disabling nature of these symptoms, even in the absence of overt cognitive dysfunction. Cumulative disability related to visual hallucinations was previously reported to significantly impact the quality of life of both patients with PD and caregivers (Weil et al., 2020). In addition to their association with cognitive decline and reduced functional independence, hallucinations are linked to increased mortality, and they are the strongest predictor of earlier nursing home placement, independently from disease duration and disease severity (Goetz and Stebbins, 1995).

## 6 Limitations

We acknowledge some potential limitations to this study, mostly inherent to its exploratory nature. Among these, patients’ hallucinatory status was determined by means of an Italian translation of the UMPDHQ. While this tool was shown to provide an accurate characterization of hallucinatory percepts occurring in English-speaking PD patients, its Italian translation remains to be validated. In addition, an extensive neuropsychological assessment of participants was not conducted, potentially limiting the interpretability of our findings. Finally, correction for multiple comparison analysis and correlation between clinical, perceptual, and brain imaging variables were not performed. An extension of the present study conducted on a larger sample size corrected for multiplicity, with correlations across a broader range of neuropsychological and perceptual variables is warranted.

## 7 Conclusion

Illusory biases relying on adequate attentional modulation and global visual perception appear to be paradoxically attenuated in PD patients without hallucinations. This pattern is not recognizable in hallucinating PD patients. Conversely, illusory percepts counteracted by proper perceptual grouping, figure to background segregation, and recognition of overlapping figures seem enhanced in PD patients with history of hallucinations. Overall, these findings suggest that the impairment of high order visuoperceptual functions occurring in PD is linked to different profiles of vulnerability to illusory biases. Computer-generated illusory figures are non-invasive, reproducible, and relatively inexpensive. Perceptual performance on these tasks could be a suitable tool to systematically characterize the neural underpinnings of hallucinations in these patients and complement available neuropsychological and clinical scales for a prompt detection and characterization of these phenomena. In our experience, these tasks can be administered to non-demented patients with mild to moderate PD with nominal time requirements and ease of recruitment.

## Data availability statement

De-identified raw data supporting the conclusions of this article will be made available upon motivated request by the authors, without undue reservation.

## Ethics statement

The studies involving humans were approved by Comitato Etico Unico Regionale FVG (CEUR). The studies were conducted in accordance with the local legislation and institutional requirements. The participants provided their written informed consent to participate in this study.

## Author contributions

AC: Conceptualization, Data curation, Formal analysis, Investigation, Methodology, Writing – original draft, Supervision. CM: Data curation, Formal analysis, Investigation, Writing – review & editing. MC: Investigation, Supervision, Writing – review & editing. ML: Investigation, Writing – review & editing. LA: Supervision, Writing – review & editing. TL: Investigation, Writing – review & editing. VC: Investigation, Writing – review & editing. SR: Data curation, Investigation, Writing – review & editing. SM: Conceptualization, Software, Writing – review & editing. CC: Writing – review & editing. FD: Writing – review & editing. MM: Conceptualization, Supervision, Writing – review & editing. TA: Conceptualization, Supervision, Writing – review & editing. PM: Conceptualization, Supervision, Writing – review & editing.
