# Supplementary material for: Using illusions to understand hallucinations: differences in perceptual performances on illusory figures may underscore specific visuoperceptual impairments in Parkinson’s disease
Source: Front Neurosci. 2023 Dec 6;17:1256224. doi: 10.3389/fnins.2023.1256224 (PMC10732246; doi:10.3389/fnins.2023.1256224)
Supplement: Supplementary file 1 [file Data_Sheet_1.PDF]

Supplemental Materials: Italian Translation of The University of Miami Parkinson's disease Hallucinations Questionnaire (UM-PDHQ).

QUESITO	CARATTERISTICHE	PUNTEGGIO
1. Le capita mai di avere delle allucinazioni?<br><br>*(Ha mai notato qualcosa di strano nella sua vista? Le è mai capitata qualche esperienza visiva insolita? Le è mai capitato di vedere, udire, sentire, odorare o gustare cose che non sono realmente presenti o che comunque gli altri non vedono?)*	1. Visive<br><br>2. Acustiche<br><br>3. Somatiche/ cutanee<br><br>4. Gustative<br><br>5. Olfattorie	0 = nessuna allucinazione<br><br>1 = un solo tipo di allucinazione<br><br>2 = combinazione di più tipi<br><br>C: non nell'ultimo mese, ma è capitato in passato.
2. Quante volte le capita di sperimentare allucinazioni?		0 = solo raramente<br><br>1 = occasionalmente (meno di una volta alla settimana ma in modo protratto)<br><br>2 = spesso (circa una volta alla settimana)<br><br>3 = frequentemente (molte volte alla settimana ma meno di una volta ogni giorno)<br><br>4 = molto frequentemente (una o più volte ogni giorno)
3. Di solito quanto durano queste esperienze?		0 = breve durata (<1 secondo)<br><br>1 = durata media (< 10 secondi)<br><br>2 = durata prolungata (>10 secondi)
4. Ritiene che ciò che le capita di vedere o sperimentare sia reale?		0 = non e´reale<br><br>1 = talvolta penso sia reale<br><br>2 = penso sempre sia reale
5. Quanti tipi di immagini o sensazioni le capita di sperimentare?		1 = sempre lo stesso tipo<br><br>2 = pochi tipi (due o tre)<br><br>3 = molti tipi (più di tre)
6. Quanto gravi, disturbanti o stressanti le paiono queste visioni o sensazioni?		0 = nessun effetto negativo o addirittura piacevoli<br><br>1 = lievemente stressanti<br><br>2 =moderatamente stressanti (infastidiscono e sono intrusive)<br><br>3 = gravemente stressanti (molto disturbanti, possono richiedere trattamento farmacologico).
**Punteggio (min 0 / max 14)**		

SEGNI LA RISPOSTA PIU' APPROPRIATA ED EVENTUALMENTE DESCRIVA	
7. Le è mai stato diagnosticato un problema agli occhi?<br><br>(es. problemi di vista, visione doppia, cataratta, glaucoma, retinite, distacco di retina, retinopatia diabetica o ipertensiva)	**SI (descriva): ______________________________**<br><br>**NO**
8. Che medicine assume?	**COMPILARE NELLA SCHEDA RACCOLTA DATI**
9. Ha modificato medicine recentemente?	**SI (descriva): ______________________________**<br>**NO**
**10.** Il cambiamento delle medicine è stato dovuto alla comparsa o a cambiamenti nelle sue allucinazioni?	**SI**<br>**NO**<br>**NON SAPREI**
11. Le allucinazioni capitano durante fasi di ON o di OFF?	**SOPRATTUTTO IN ON**<br><br>**SOPRATTUTTO IN OFF**<br><br>**SEMPRE, A PRESCINDERE DA ON E OFF**
12. Di solito cosa le capita di vedere?	**LE ALLUCINAZIONI NON HANNO FORMA, NON SAPREI DESCRIVERLE**<br><br>**VOLTI:**<br>**a. interi**<br>**b. frammentati b1. familiari / b2 estranei**<br><br>**PERSONE INTERE:**<br>**a. familiari**<br>**b. estranee**<br><br>**ANIMALI**<br><br>**INSETTI / RETTILI**<br><br>**OGGETTI**
13. C'è qualcosa che può fare cessare queste immagini/sensazioni?	**SI (descriva): _____________________________**<br><br>**NO**
14. In che momento del giorno o con quali condizioni di luminosità si verificano di solito?	**IN MOMENTI SPECIFICI DEL GIORNO**<br>- **Di giorno / in piena luce**<br>- **Di notte / nell'oscurità**<br>- **Al crepuscolo**<br><br>**SI VERIFICANO IN QUALSIASI MOMENTO**
15. Le immagini che vede producono qualche suono o rumore?	**SI**<br><br>**NO**<br><br>**N/A (assenza di allucinazioni visive)**

16. Le immagini che vede si muovono?	**SI**<br><br>**NO**<br><br>**N/A (assenza di allucinazioni visive)**
17. Le immagini che vede hanno dimensioni normali?	**SI, sono piu' piccole del normale**<br><br>**SI, sono piu' grandi del normale**<br><br>**NO**<br><br>**N/A (assenza di allucinazioni visive)**
18. Le immagini che vede sono evanescenti o solide?	**EVANESCENTI**<br><br>**SOLIDE**<br><br>**N/A (assenza di allucinazioni visive)**
19. Le immagini che vede sono colorate?	**SI**<br><br>**NO (bianche e nere)**<br><br>**N/A (assenza di allucinazioni visive)**
20. Le immagini che vede compaiono in modo graduale o improvviso?	**GRADUALE (compaiono e scompaiono lentamente)**<br><br>**IMPROVVISO (compaiono e scompaiono improvvisamente)**<br><br>**NON SAPREI**<br><br>**N/A (assenza di allucinazioni visive)**

DISCLAIMER: This is an original, non-validated translation developed by Alberto Cucca M.D. for the Department of Life Sciences of the University of Trieste for the sole scientific purposes of the present study. For further information: alberto.cucca@phd.units.it.

NOTA: Questa traduzione originale e non validata in Italiano è stata sviluppata dal Dott. Alberto Cucca per il Dipartimento di Scienze della Vita dell'Università degli Studi di Trieste a scopi unicamente sperimentali e limitatamente alla sperimentazione in oggetto. Per informazioni, alberto.cucca@phd.units.it.
